# Elemental Boron Nanoparticles for Boron-Neutron Capture Therapy of BT-474 Human Breast Cancer

**DOI:** 10.3390/nano16160973

**Published:** 2026-08-07

**Authors:** Evgenii L. Zavjalov, Linga D. Romanenko, Olga I. Kichakova, Anna I. Kasatova, Polina A. Kotelnikova, Dmitry S. Petrunya, Ekaterina V. Barmina, Kuder O. Aiyyzhy, Artem A. Laktionov, Sergei M. Klimentov, Anton A. Popov, Maria S. Grigoryeva, Timofey A. Bykov, Vasilisa V. Podolyako, Anastasia A. Fronya, Egor I. Mavreshko, Danila A. Pokhorukov, Sergey Yu. Taskaev, Sergey M. Deyev, Irina N. Zavestovskaya

**Affiliations:** 1 Institute of Cytology and Genetics, Siberian Branch of the Russian Academy of Sciences, Novosibirsk 630090, Russia; 2 P.N. Lebedev Physical Institute of the Russian Academy of Sciences, Moscow 119333, Russia; yarullinaai@yahoo.com (A.I.K.); d.petrunya@lebedev.ru (D.S.P.); e.mavreshko@lebedev.ru (E.I.M.);; 3 Budker Institute of Nuclear Physics, Siberian Branch of the Russian Academy of Sciences, Novosibirsk 630090, Russia; 4 Novosibirsk State University, Novosibirsk 630090, Russia; 5 Shemyakin-Ovchinnikov Institute of Bioorganic Chemistry, Russian Academy of Sciences, Moscow 117437, Russiabiomem@mail.ru (S.M.D.); 6 National Research Centre “Kurchatov Institute”, Moscow 123182, Russia; 7 Prokhorov General Physics Institute, Russian Academy of Sciences, Moscow 119991, Russia; aiyyzhy@phystech.edu; 8 Institute of Engineering Physics of Biomedicine, National Research Nuclear University “MEPhI”, Moscow 115409, Russia; aalaktionov@mephi.ru (A.A.L.);

**Keywords:** boron neutron capture therapy (BNCT), elemental boron particles (BPs), mice, SCID, xenografts, BT-474, breast cancer, in vivo, in vitro, HER2, Affibody

## Abstract

**Summary:**

BNCT is capable of selectively destroying tumor cells and is a promising treatment for HER2-positive breast cancer. The success of the therapy depends on the effective delivery of boron to cancer cells. Here, we present the elemental boron nanoparticles (BPs) generated by laser fragmentation, coated with Silane-PEG-COOH and conjugated with the Affibody _ZHER2:342_ guide protein, along with the results of their application for BNCT against the HER2-positive breast cancer. In vitro results with BT-474 human breast cancer cells demonstrated high efficacy synthesized BPs in BNCT. The experiments using female SCID mice bearing BT-474 xenografts also revealed high BNCT efficacy BPs. Thus, the results demonstrate the high potential of the laser-synthesized elemental boron nanoparticles as a promising platform for boron delivery into HER2-positive tumors for BNCT.

**Abstract:**

Breast cancer remains one of the most common malignancies worldwide. Highly invasive HER2-positive breast cancer carries a high risk of metastasis and poses a significant therapeutic challenge. Boron neutron capture therapy (BNCT) may be an option for patients with this severe diagnosis. Here we evaluated the effectiveness of BNCT with elemental boron nanoparticles obtained by laser fragmentation and coated Silane-PEG-COOH and conjugated with the Affibody _ZHER2:342_ guide protein (BPs) against the HER2-positive breast cancer. The MTT assay after BNCT with BPs revealed an almost twofold reduction in surviving BT-474 tumor cells compared to the control group. The results of the clonogenic test showed totally death of BT-474 cells after BNCT with BPs at a concentration of 40 μg/mL in the culture medium. The same results was found for in vivo. In female SCID mice bearing BT-474 breast tumor xenografts, BNCT with intratumoral injection of BPs at a dose of 60 mg/kg led to a significant slowdown in xenograft growth beginning on the 17th day compared with control animals and the 39th day compared with irradiated females. A single intratumoral administration of BPs at a dose of 60 mg/kg did not show toxic effects. Histological examinations did not reveal systemic accumulation of the studied BPs in major organs; instead BPs was selectively retained within the xenografts and surrounding tissues. Thus, laser fragmented functionalized with Silane-PEG-COOH and the Affibody _ZHER2:342_ (BPs B-PEG-AFF) represent a promising platform for boron delivery in targeted BNCT applications.

## 1. Introduction

Despite ongoing efforts, cancer remains one of the leading causes of death worldwide. Breast cancer (BC) is one of the most common tumor types in women [1]. In 2020 alone, over 2.3 million new cases of BC were reported worldwide [2]. At the molecular level, breast cancer is a heterogeneous disease involving activation of the human epidermal growth factor receptor 2 (HER2, encoded by the *ERBB2* gene), activation of hormonal receptors (estrogen receptor and progesterone receptor), and/or mutations in the *BRCA* genes—factors that in turn determine treatment strategies [3]. Overexpression of the HER2 protein in BC tumor cells is classified as HER2-positive breast cancer [4]. This subtype is characterized by high invasive and metastatic potential, resulting in an unfavorable prognosis for patients [1]. The HER2-positive subtype accounts for only 20% of all BC cases but remains the most challenging subtype to treat in clinical practice [5]. Several HER2-targeted agents are currently used in clinical practice, including monoclonal antibodies such as trastuzumab and pertuzumab, antibody–drug conjugates such as ado-trastuzumab emtansine (T-DM1) and trastuzumab deruxtecan, and small molecule tyrosine kinase inhibitors [5,6]. However, their efficacy can be limited by intrinsic or acquired resistance, intratumoral heterogeneity, changes in HER2 expression, and rapid tumor evolution under therapeutic pressure. Thus, there is a need for alternative therapeutic approaches that retain tumor-specific targeting while exerting potent cytotoxic effects through mechanisms that bypass conventional drug-resistance pathways.

Currently, research is underway to develop BNCT approaches for the selective destruction of tumor cells, including breast cancer [7,8]. HER2-directed boron neutron capture therapy (BNCT) may address this need by combining selective delivery of ^10^B-enriched agents to HER2-positive cells with intracellular neutron-capture reactions that generate high-LET particles and highly localized, difficult-to-repair DNA damage. Boron delivery to tumor cells can be achieved in various ways, including targeted drugs based on antibodies or ligands against tumor cell receptors [9], boronated amino acids (boronophenylalanine, BPA) or carboxylic acid salts (sodium mercaptoborate, BSH), and liposomes [10,11,12,13]. Despite the successful use of these boron delivery systems in tumor cells, achieving the necessary boron concentrations for effective BNCT often remains challenging. Furthermore, the selection of a boron-containing compound for BNCT is complicated by specific requirements: high tumor affinity to achieve the target concentration in the tumor (>20 μg/g tissue) and prolonged retention in tumor cells (>1 h), while simultaneously maintaining low systemic toxicity and minimal accumulation in healthy tissues (tumor-to-healthy tissue ratio of 3:1) [14]. In this context, the use of elemental boron particles (BPs) modified with biocompatible coatings is considered a promising direction for BNCT due to the high density of boron atoms (~10^2^–10^5^ atoms per particle), the high potential for passive accumulation in the tumor via the EPR (enhanced permeability and retention) effect, and the possibility of their active delivery to the tumor by functionalizing their surface with targeted molecules [8,15,16].

Studies on the accumulation of boron and the antitumor efficacy of BNCT in combination with boron-containing nanostructures have revealed the significant potential of these agents [17,18]. It has been shown that the survival rate of human glioblastoma cells in vitro after neutron beam irradiation with the addition of BPs was reduced to almost 0%. This reduction was significantly greater than that observed in the groups irradiated without BPs or with BPA [7]. The efficacy of BNCT in vivo in combination with boron carbide nanoparticles was demonstrated in female BALB/c mice. In animals with heterotopic grafts of CT26 colon carcinoma cells, the group that received BNCT with intravenous administration of boron carbide nanoparticles showed the smallest tumor sizes [19].

The aim of this study was to investigate the efficacy of BNCT using in vitro and in vivo models of BT-474 HER2-positive human breast cancer in combination with boron nanoparticles obtained by laser fragmentation of crystalline elemental boron samples.

## 2. Materials and Methods

### 2.1. Laser Synthesis of Boron Particles

The elemental boron nanoparticles (BPs) were obtained by laser fragmentation of boron-10 micropowder within a flow cell containing isopropanol. A Nd:YAG laser (EKSPLA NL210-SH, Ekspla, Vilnius, Lithuania) with a wavelength of 1064 nm, a pulse duration of 4 ns, a pulse energy of 2.8 mJ, and a pulse repetition rate of 1 kHz was used. The boron concentration was 100 µg/mL, and the volume of the working fluid was 70 mL. The laser beam was moved using a galvano-optical system at a speed of 100 mm/s and focused using an F-theta lens (focal length 10 cm). The estimated diameter of the laser beam at the focal point was 50 μm. The fragmentation time of the suspension was 60 min. Images of the BPs were obtained using a scanning electron microscope (MAIA 3, Tescan, Brno, Czech Republic) at an accelerating voltage of 30 keV.

### 2.2. Surface Modification and Characterization of Boron Particles

The surface of the BPs was modified with silane-polyethylene glycol with carboxyl groups (Silane-PEG-COOH, 5 kDa) using silane chemistry methods to stabilize the colloidal solutions of particles and introduce functional groups for subsequent conjugation [20]. To 1 mg of BPs in 1 mL of ethanol, 65 μL of water and 20 μL of 30% ammonium hydroxide were added per 1 mg of BPs, in 1 mL of ethanol. The mixture was treated in an ultrasonic bath, followed by the addition of 100 μL of a Silane-PEG-COOH solution (1 g/L) in ethanol. The mixture was ultrasonicated and incubated at 60 °C for 2 h and then at 25 °C for 16 h. The PEG-coated particles were washed three times with ethanol and deionized water by centrifugation at 20,000× *g* for 15 min.

Changes in particle size and zeta potential were assessed using a Zetasizer Nano ZS analyzer (Malvern Instruments, Malvern, UK).

FTIR spectra were measured in the range of 4000–500 cm^−1^ using an FT-801 spectrometer (Simex, Novosibirsk, Russia), which has a diamond thermal cell of frustrated total internal reflection. To denoise the signal, the resulting spectra were smoothed using Savitzky–Golay method with a window range of 10 and polynomial order of 2.

### 2.3. Production and Isolation of Recombinant Affibody Protein

The recombinant Affibody _ZHER2:342_ protein was produced by auto-induction in BL21 (DE3) *E. coli* cells, followed by purification using metal-affinity chromatography on HisTrap™ HP columns (Cytiva, Sweden AB, Uppsala, Sweden) with a stepwise gradient of imidazole (50–250 mM) in phosphate buffer, as described previously [21,22]. The fractions were analyzed by denaturing SDS-PAGE using a Tris-Tricine system. Before conjugation, the protein was transferred to the required buffer solution by gel filtration.

### 2.4. Conjugation of Boron Particles with Biomolecules

Recombinant Affibody _ZHER2:342_ proteins were attached to the surface of boron particles using the carbodiimide method. B-PEG-COOH particles were incubated with 1-ethyl-3-(3-dimethyl aminopropyl)carbodiimide (EDC) and N-hydroxysulfosuccinimide (sulfo-NHS) (Thermo Fisher Scientific, Waltham, MA, USA) in 10 mM 2-(N-morpholino) ethanesulfonic acid (MES) buffer (pH 5.5) for 30 min at room temperature to activate the carboxyl groups. Next, the BPs were purified by centrifugation from unreacted components and resuspended in 50 mM HEPES buffer (pH = 7.5), and Affibody _ZHER2:342_ was added. The mixture was incubated for 2 h at room temperature, and then the particles were washed three times to remove unbound protein by centrifugation. The attachment efficacy of the Affibody _ZHER2:342_ to the surface of BPs was confirmed by changes in the hydrodynamic size and zeta potential of the particles using a Zetasizer Nano ZS analyzer (Malvern Instruments, Malvern, UK).

### 2.5. MTT and Clonogenic Tests

BT-474 (ATCC HTB-20™) HER2-positive human breast carcinoma cell line was obtained from Lobachevsky State University of Nizhny Novgorod, Russia. Cell were cultured in RPMI-1640 medium (Biolot, Saint Petersburg, Russia) supplemented with 10% FBS (Capricorn, Ebsdorfergrund, Germany). Four experimental groups were used: control (no BPs, no irradiation), irradiation only (neutron flux without BPs), “BPs” (BPs only, no irradiation) and “BPs + BNCT” (pre-incubation with BPs followed by irradiation). Cells were seeded in culture flasks with a surface area of 25 cm^2^, and after 24 h, the cells were placed in a medium containing BPs at a concentration of 40 μg/mL complete culture medium and incubated for 24 h. Before irradiation, the cells were washed with sodium phosphate buffer, trypsinized, centrifuged, and transferred to a polypropylene cryopreservation tubes at a concentration of 1 × 10^6^ cells per mL of complete culture medium. The cryopreservation tubes were transported in a thermally insulated container.

To perform the MTT-test, cells from the experimental groups, both non-irradiated and irradiated, were seeded in 96-well plates at a density of 4 × 10^3^ cells per well and left for 24 h; the number of replicates for each condition was 5 (*n* = 5). Afterward, the optical density of the solutions in each well was measured using a Multiskan SkyHigh spectrophotometer (Thermo Fisher Scientific, USA). The percentage of surviving cells was calculated relative to the control. For the clonogenic assay, 200 cells were seeded per well in a 12-well culture plate, with four replicates for each experimental group (*n* = 4). On day 10, the colonies were fixed with a mixture of 6% glutaraldehyde and 0.5% crystal violet. Colonies containing more than 50 cells were counted visually using an Olympus CKX53 inverted light microscope (Olympus, Tokyo, Japan). The proportion of surviving cells in the experimental groups was calculated based on the survival rate in the control group.

### 2.6. Irradiation

**Figure 1:**
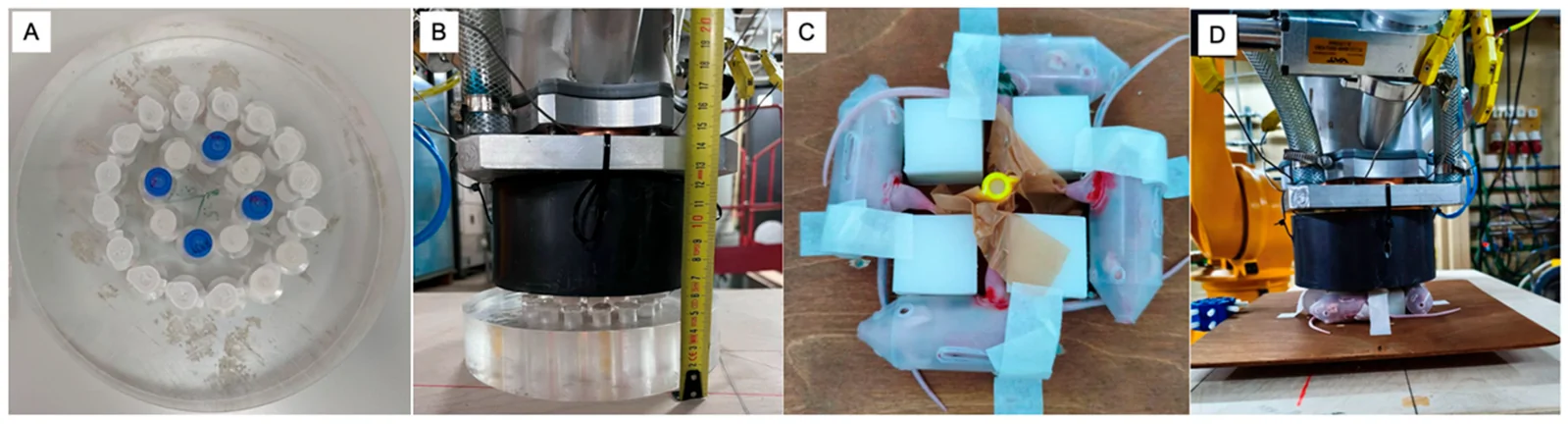
Experimental setup for irradiation of biological objects at the VITA accelerator-based neutron source: (**A**) PMMA phantom for placement of cryovials, (**B**) positioning of the PMMA phantom under the beam shaping assembly, (**C**) arrangement of mice for irradiation, (**D**) positioning of the animals under the beam shaping assembly.

Cells and animals were irradiated at the VITA accelerator neutron source [23]. The VITA accelerator neutron source consists of a tandem accelerator with vacuum insulation for producing a stationary proton or deuteron beam, an original thin lithium target for neutron generation via the ^7^Li(p,n)^7^Be reaction, and a vertical beam shaping assembly optimized for irradiation of specific biological objects. The beam shaping assembly included a 72 mm-high polyethylene block with bulk bismuth inclusions, placed between the target assembly and the biological objects. Cryovials containing cells were placed into the compartments of a 220 mm-high polymethyl methacrylate (PMMA) phantom, 25 mm from the phantom center (Figure 1A). Compartments without cell samples were filled with cryovials containing deionized water. The phantom was positioned 10 mm below the beam shaping assembly (Figure 1B). Cells were irradiated in a cryopreservation tube for 30 min immediately after the addition of BPs at the studied doses using a proton energy of 2.0 MeV to a proton fluence of 1 mA·h. For the animal experiments, mice were injected with BP suspensions 40–60 min before irradiation according to their experimental group, placed in plastic restraints with an opening for the xenograft-bearing paw, and irradiated at a proton energy of 2.1 MeV to a proton fluence of 2.5 mA·h for 90 min. For additional immobilization of the mice and further neutron moderation in the xenograft area, four polyethylene cubes were placed between the animals (Figure 1C). Four mice were irradiated per session, and the mice were positioned directly adjacent to the beam shaping assembly (Figure 1D).

### 2.7. In Vivo Studies

The study was conducted at the Center for Genetic Resources of Laboratory Animals of the Institute of Cytology and Genetics of the Siberian Branch of the Russian Academy of Sciences using 8 to 10-week-old immunodeficient female SCID mice. The animals were kept in groups of 3–5 individuals under standard conditions [10]. The housing conditions complied with the standards specified in Directive 2010/63/EU on the protection of animals used for scientific purposes. The study protocol was approved by the Bioethics Committee of the Institute of Cytology and Genetics of the Siberian Branch of the Russian Academy of Sciences (Protocol No. 254, dated 27 October 2025). BT-474 cells (ATCC HTB-20™) overexpressing the HER2 protein were used to obtain human breast cancer xenografts. After cultivation, the cells were trypsinized, centrifugated, resuspended in serum-free medium, and 100 μL of the cell suspension (2 × 10^6^ cells) was injected subcutaneously into the right thigh of the mice. Xenograft volume (*V*) was measured using a caliper and then calculated according to the formula [24]: *V* = (*a* × *b*^2^) × 0.52, where *a* is the largest tumor diameter and *b* is the smallest. The tumor growth inhibition value (*TGI*) was calculated using the formula [25]: *TGI* (%) = (*V_k_* − *V_o_*)/*V_k_* × 100, where *V_k_* is the mean volume of the xenograft in the control group, and *V_o_* is the mean volume of the xenograft in the experimental group. The animals were weighed, and the linear dimensions of the xenograft were measured every 3–4 days.

Animals with a xenograft volume reaching 70 mm^3^ were included in the experiment. A total of 4 groups were formed: control animals injected with saline, irradiated animals injected with saline, animals with intratumoral injection of BPs at a dose of 60 mg/kg and BNCT animals after intratumoral injection of BPs at a dose of 60 mg/kg. Immediately before injection, the nanoparticles suspensions were treated for 1 min at an amplitude of 20 using an ultrasonic homogenizer Qsonica Q700 (Qsonica LLC, Newtown, CT, USA). The volume of a single administration of the studied BPs suspensions was 80–100 μL depending on the body weight of the animals. Animals with a xenograft volume reaching 5000 mm^3^ were euthanized. After euthanasia, the xenografts, surrounding tissues, and organs (brain, heart, spleen, and lungs) were collected for histological analysis.

### 2.8. Histological Research

The tissues were fixed in 10% formalin for subsequent histological analysis. After hematoxylin and eosin staining, the preparations were examined using a DM 2500 light microscope (Leica, Wetzlar, Germany), and 10 fields of view were analyzed per sample.

### 2.9. Statistical Analysis of the Results

Statistical analysis was performed using STATISTICA 6.0 software. Student’s *t*-test was used to compare cell data. The Mann–Whitney U-test and ANOWA were used to compare xenograft volumes. The timing of animal withdrawal from the experiment was presented as Kaplan–Meier curves using the Cox F-test. Intergroup differences in xenograft volume were evaluated using ANOVA. The results were expressed as the mean ± standard error of the mean (M ± SEM). Differences were considered statistically significant at *p* < 0.05.

## 3. Results

### 3.1. Synthesis and Characterization of Boron Particles

**Figure 2:**
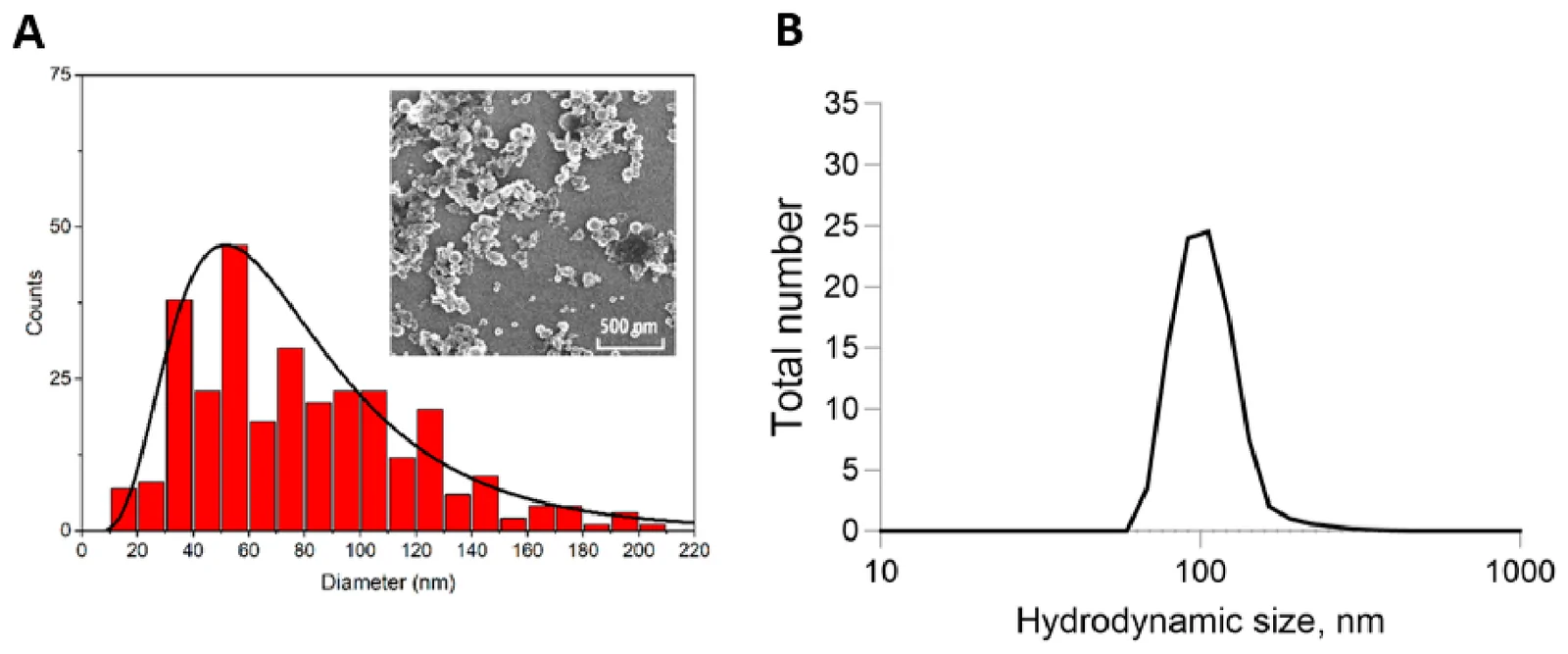
Size distribution diagrams of elemental BPs obtained by laser fragmentation along with corresponding electron microscopy images (**A**). Hydrodynamic size of BPs in aqueous medium (**B**).

Laser fragmentation of boron micropowder for 60 min resulted in the formation of BPs ranging in size from 5 to 200 nm, the majority of which fell between 20 and 100 nm (Figure 2A). Electron microscopy revealed the spherical shape of particles. In aqueous medium, the lyophilized nanoparticles exhibited a hydrodynamic diameter of 107 ± 30 nm with a polydispersity index (PDI) of 0.232 (Figure 2B).

**Figure 3:**
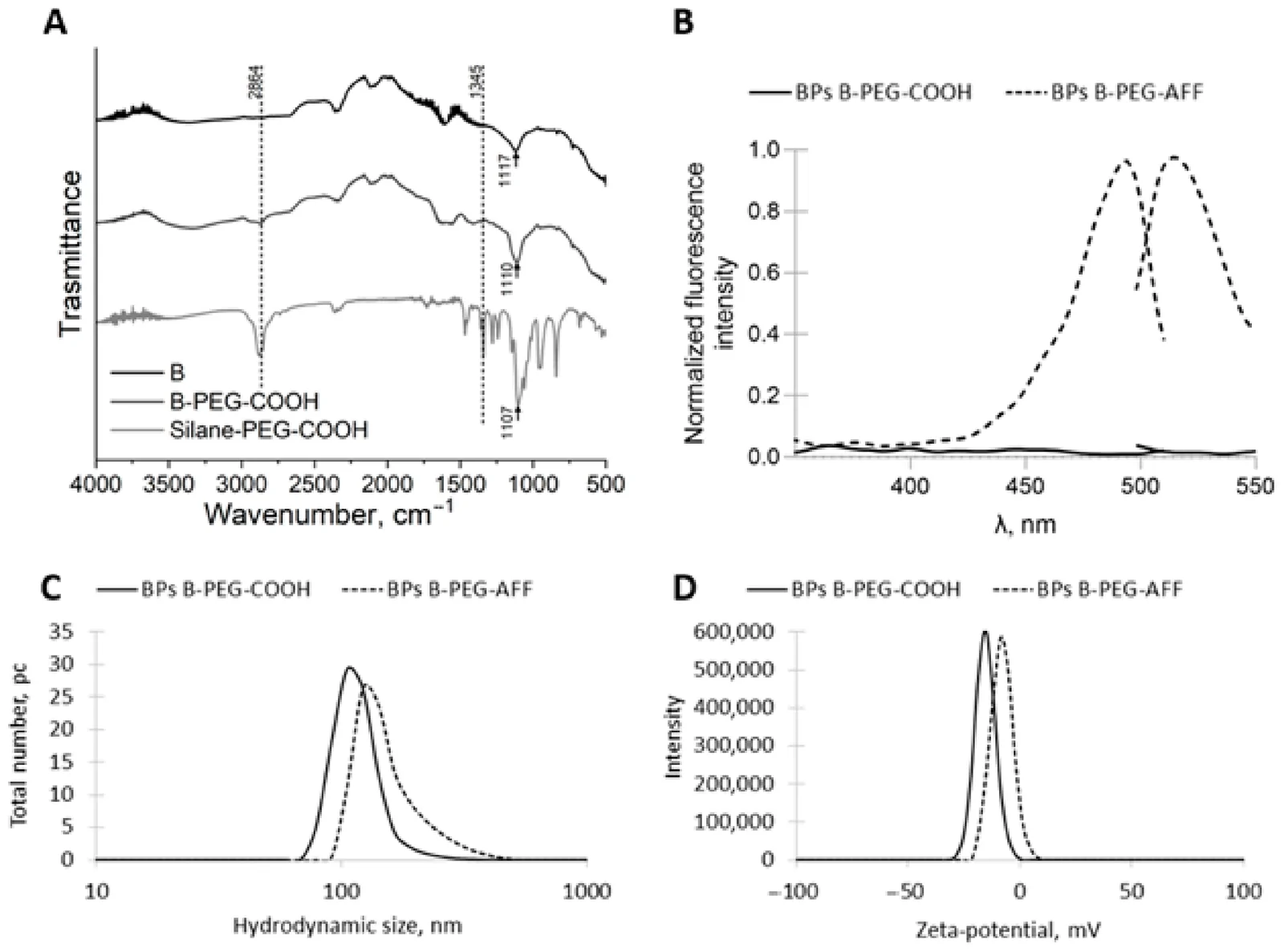
Fluorescence spectra of pristine nanoparticles and nanoparticles conjugated with AF488-labeled Affibody (**A**). FTIR spectra of pristine boron nanoparticles and carboxylated PEGylated boron nanoparticles (B-PEG-COOH), compared with silane-PEG-COOH (**B**). Hydrodynamic size of BPs (**C**) modified with Silane-PEG-COOH, before and after conjugation with the Affibody _ZHER2:342_, and their zeta potential values (**D**).

To improve colloidal stability and provide reactive carboxyl groups for subsequent surface functionalization, the synthesized boron nanoparticles were coated with silane-PEG-COOH. The FTIR spectra of the nanoparticles confirmed successful PEGylation of the particle surface. The coated B NPs exhibited additional vibrational bands characteristic of PEG, mainly C–H stretching (2864 cm^−1^) and asymmetric C–O–C stretching (1345 cm^−1^). Moreover, the band initially observed at 1117 cm^−1^ shifted to 1110 cm^−1^ after coating, approaching the characteristic PEG absorption at 1107 cm-1, further supporting successful surface functionalization (Figure 3).

Affibody _ZHER2:342_, which binds the HER2 receptor with a dissociation constant of 22 pM, was chosen as a targeting module [26]. The protein was covalently attached to the nanoparticle surface using carbodiimide-mediated conjugation. After attachment of the positively charged Affibody _ZHER2:342_, the zeta potential of BPs changed from −15.6 ± 4.7 mV to −8.1 ± 4.9 mV. After conjugation with the Affibody _ZHER2:342_, the hydrodynamic size of BPs increased from 118 ± 30 nm to 160 ± 58 nm. Successful protein conjugation was confirmed using AF488-labeled Affibody. After conjugation, the fluorescence spectra of the nanoparticles displayed AF488-characteristic excitation and emission peaks, which were not observed for pristine nanoparticles (Figure 3).

**Figure 4:**
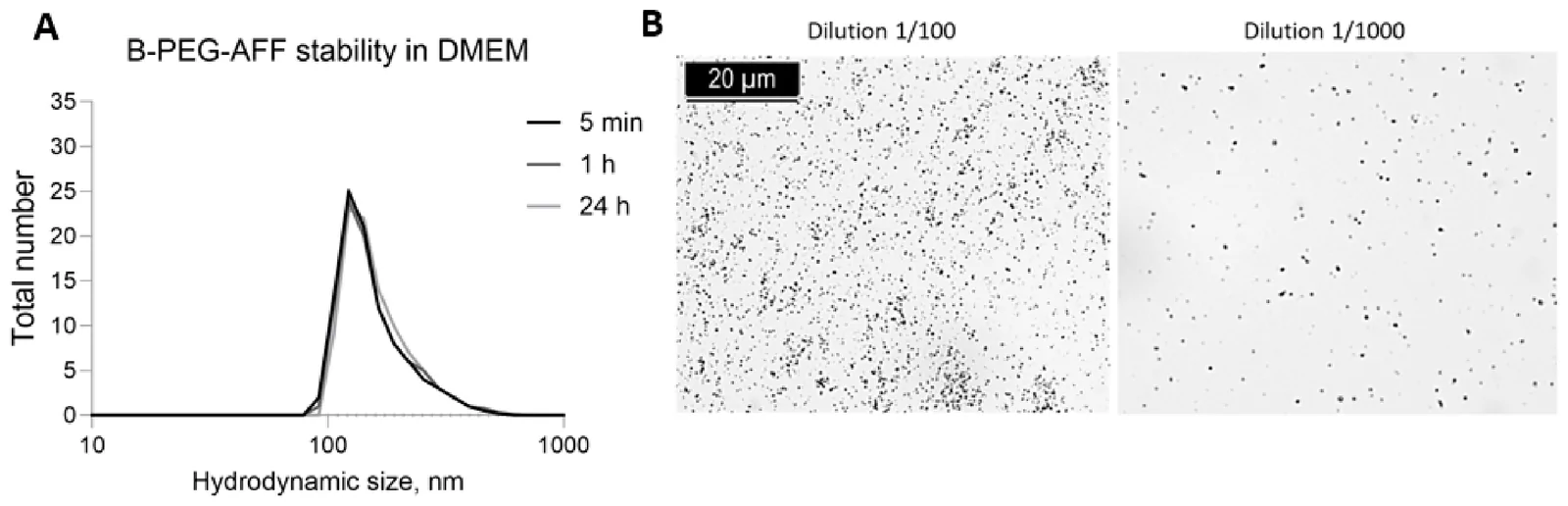
Time-dependent hydrodynamic size distribution profiles of nanoparticles in DMEM supplemented with 10% FBS, demonstrating their colloidal stability in cell culture medium (**A**). Microphotographs of BPs at 100-fold and 1000-fold dilutions. Magnification: ×1000 (**B**).

The particles remained stable in water and culture medium for at least 24 h, corresponding to the nanoparticle–cell exposure time used in this study. Measurement of the hydrodynamic size of the nanoparticles in DMEM supplemented with 10% FBS showed that the particle size remained largely unchanged after 5 min, 1 h, and 24 h of incubation, with only a slight broadening of the size distribution (Figure 4). The hydrodynamic diameters were 177 nm (PDI = 0.110), 162 ± 85 nm (PDI = 0.110), and 168 ± 93 nm (PDI = 0.146), respectively. A study of the nanoparticles diluted 100-fold did not showed an aggregated microstructure and only single particles were observed at a 1000-fold dilution.

### 3.2. In Vitro Studies

**Figure 5:**
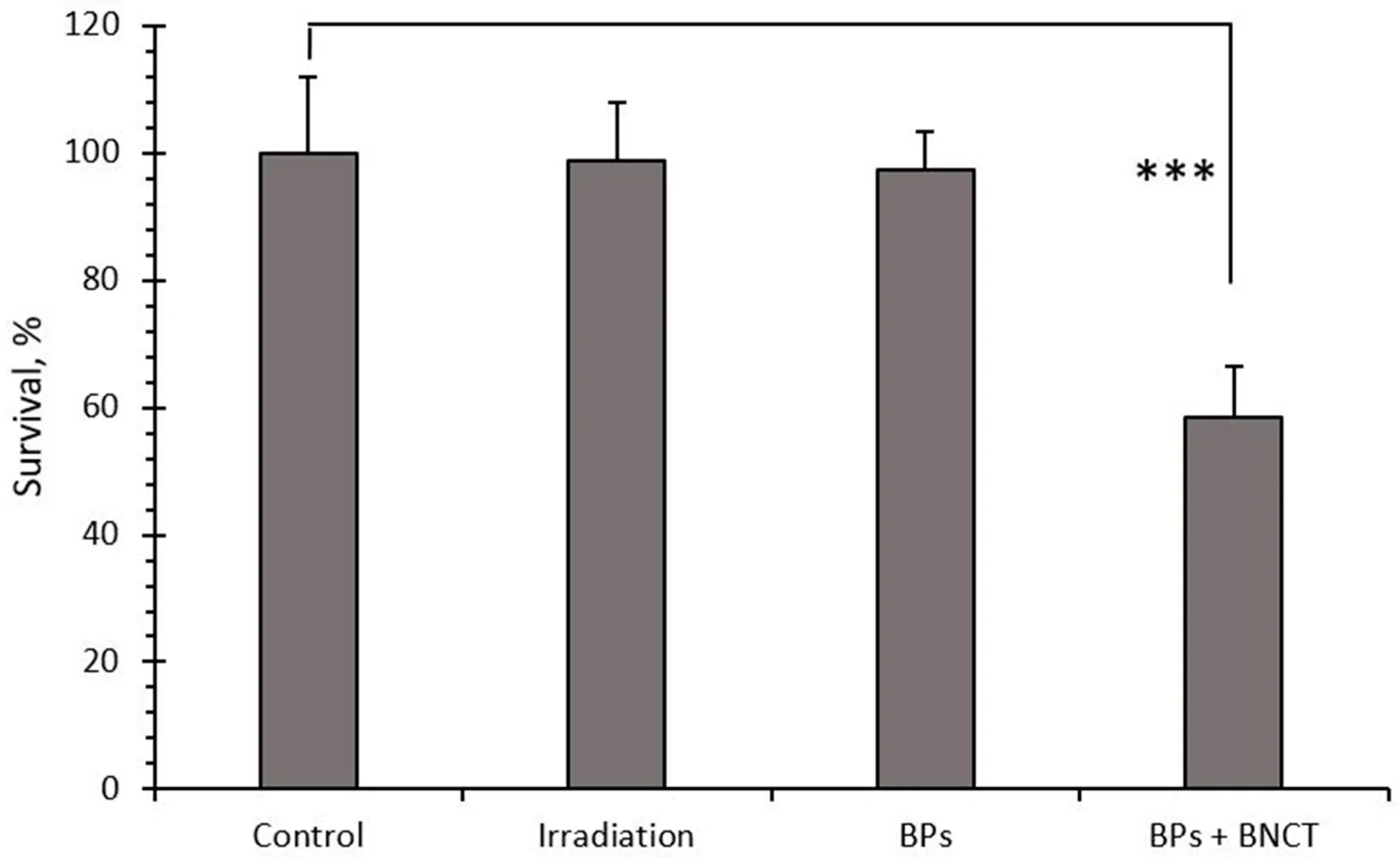
Viability of BT-474 human breast tumor cells in the MTT test after 24 h of incubation with the studied BPs. ***—*p* < 0.001, BNCT vs. control. Student’s *t*-test.

The addition of BPs at a concentration of 40 μg/mL to the culture medium exhibited no toxic effects on BT-474 cells (Figure 5). However, after 24 h of incubation, BNCT with the nanoparticles significantly reduced the tumor cell survival rate to 59% (t = 7.71, *p* = 0.00001).

The clonogenic assay for BT-474 cells (Table 1) showed a significant decrease in the survival and colony-forming ability after the addition BPs (t = 5.05, *p* = 0.007) to the culture medium. BNCT led to almost complete BT-474 cell death (t = 15.1, *p* = 0.0001).

### 3.3. In Vivo Studies

**Figure 6:**
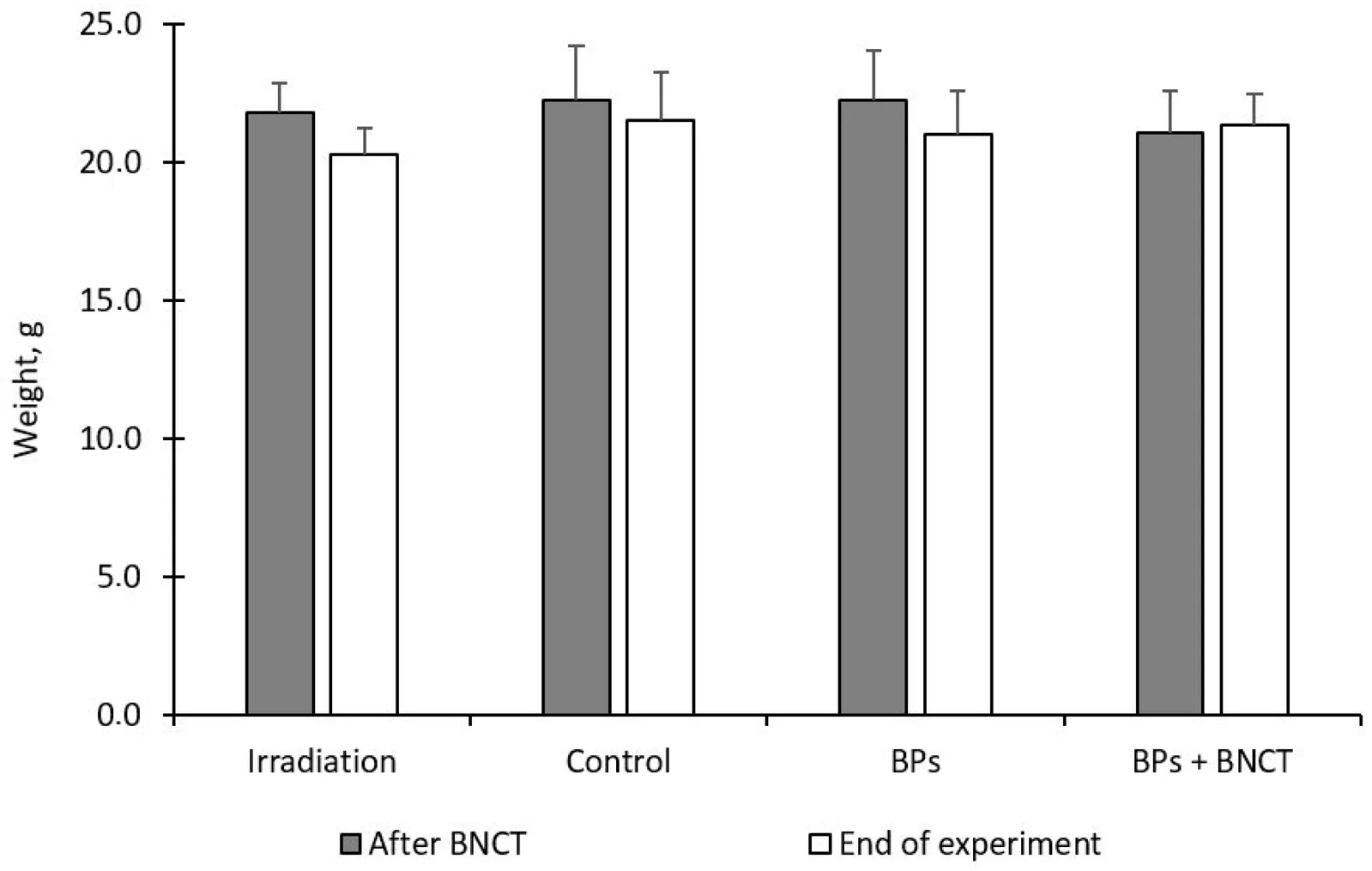
Changes in body weight of female SCID mice with xenografts of BT-474 human breast tumor cells after BNCT with intratumoral injection of BPs.

The body weight of the animals did not change throughout the experiment (Figure 6). Radiation did not significantly affect animal body weight.

**Figure 7:**
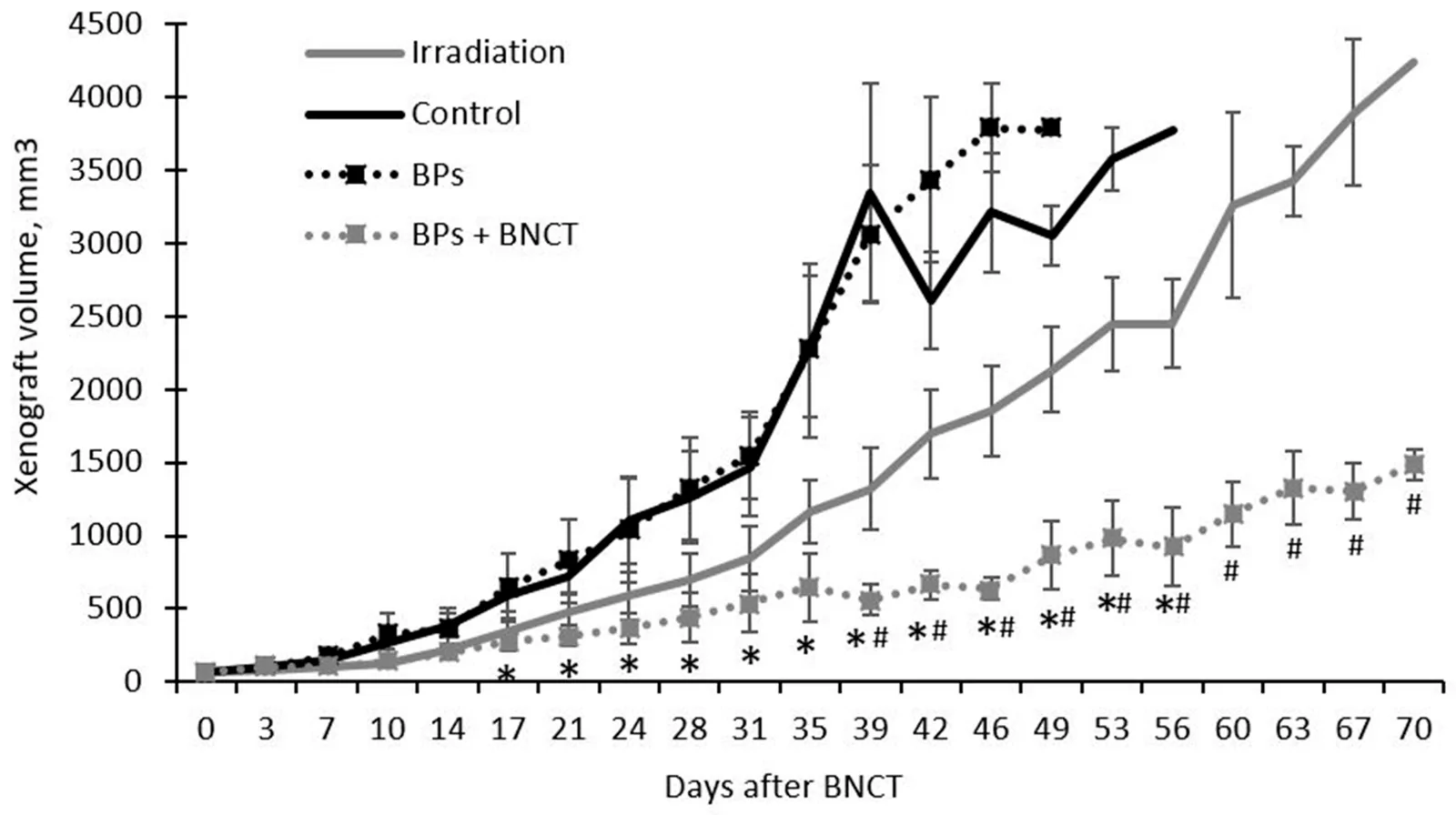
Dynamics of xenograft growth of BT-474 human breast tumor cells in female SCID mice after BNCT with intratumoral injection of the studied BPs. *—*p* < 0.05, BNCT vs. control; #—*p* < 0.05, BNCT vs. irradiation. Mann–Whitney U-test.

Xenografts in mice from different groups showed similar growth dynamics and size during the first three weeks after BNCT (F_3,23_ = 2.83, *p* = 0.06) (Figure 7). However, the TGI value differed between the groups with and without irradiation from the first week after treatment (Table 2). The administration of the BPs even had a stimulating effect on xenograft growth, while BNCT with the nanoparticles slowed tumor growth from the first week after irradiation. The reduction in xenograft growth on day 39 in BPs + BNCT mice led to significant differences in xenograft volume compared to the irradiation group (Figure 7). The TGI value in this group was the highest among all groups (78%), whereas in the irradiation group it was only 49% (Table 2). From day 39 until the end of the experiment, the size of xenografts in mice with BPs and BNCT was significantly lower than in all other groups (F_3,23_ = 7.75, *p* = 0.0009) (Figure 7).

**Figure 8:**
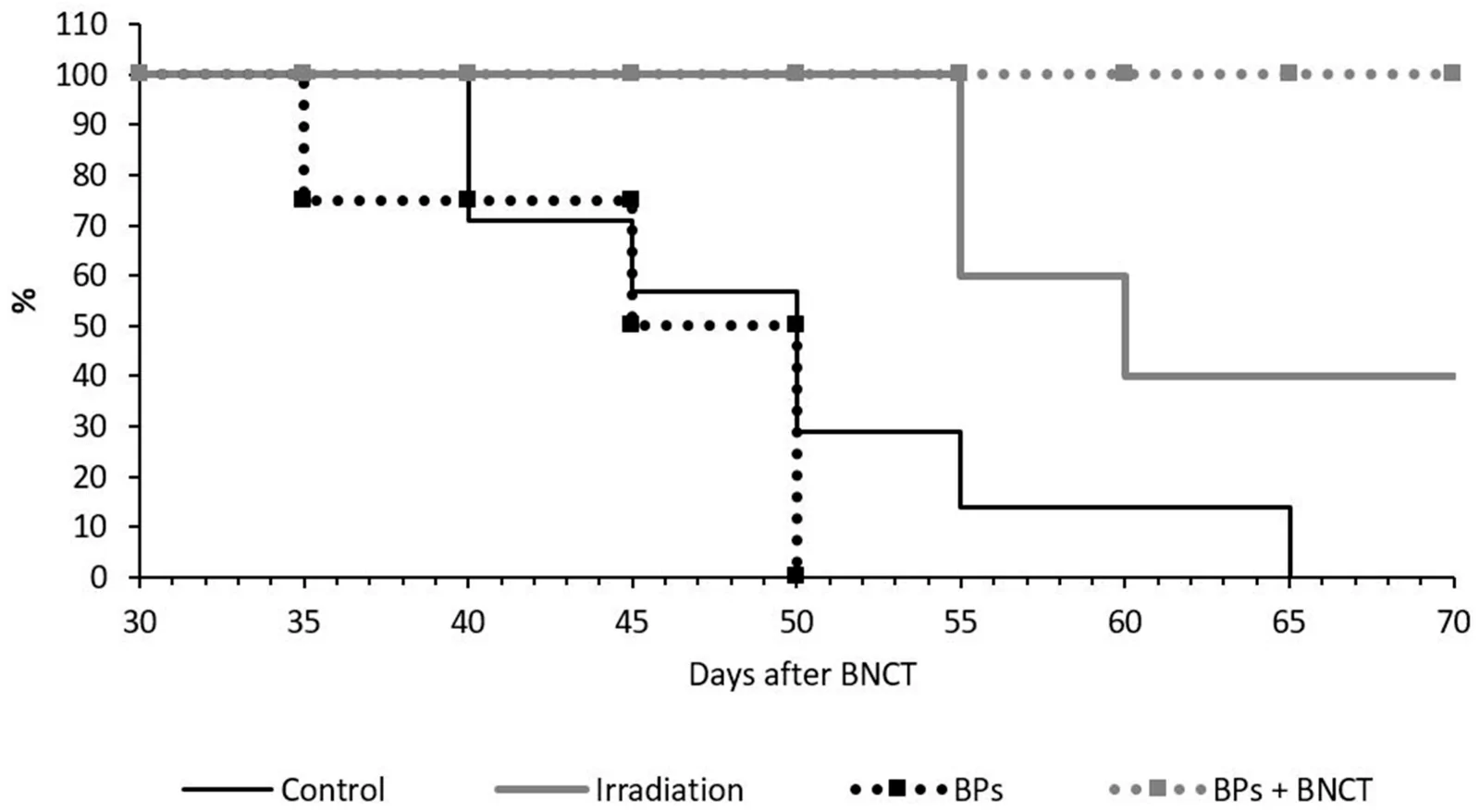
Kaplan–Meier curves for female SCID mice bearing BT-474 human breast tumor xenografts after BNCT with intratumoral injection of the studied BPs.

The animals with xenografts that reached a critical size were removed from the experiment. Based on these data, Kaplan–Meier curves were constructed (Figure 8). Mice treated only by BPs were removed from the experiment earlier than others on day 50 after BNCT. In the same group, the first animals were euthanized on day 35. In the control group almost all animals were removed on day 55, with the exception of one female removed on day 65 after the start of the experiment. In the irradiation group, 60% of mice were removed on day 70 of the experiment (F_10,14_ = 3.16, *p* = 0.03 vs. control), while none of the mice from the BNCT group were removed throughout the entire experiment. This outcome differed significantly from the irradiation group (F_16,10_ = 2.42, *p* = 0.08) and the control group (F_16,14_ = 5.10, *p* = 0.002).

**Figure 9:**
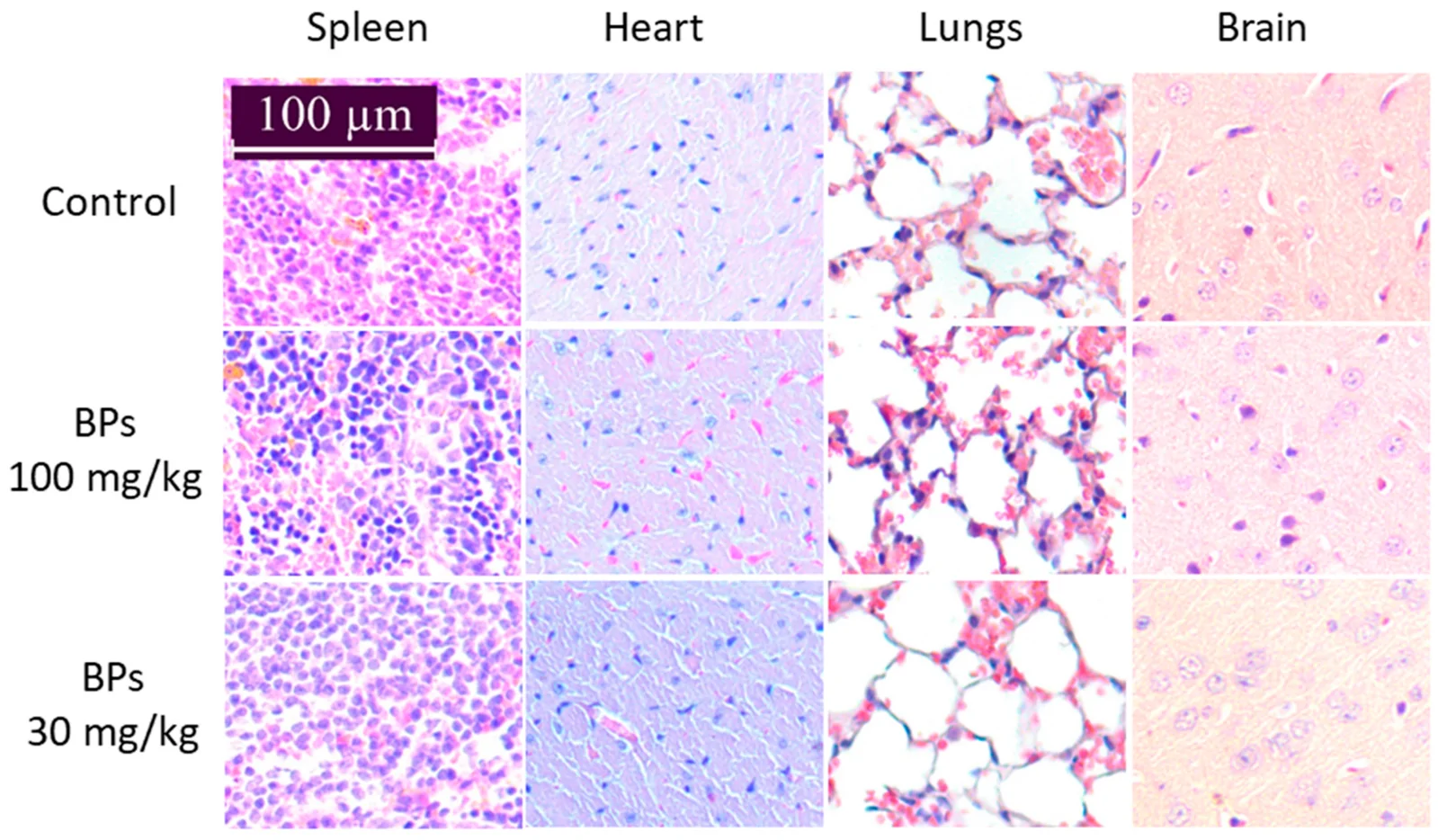
Microstructure of the studied organs in female SCID mice after intratumoral injection of the BPs. Magnification: ×200.

**Figure 10:**
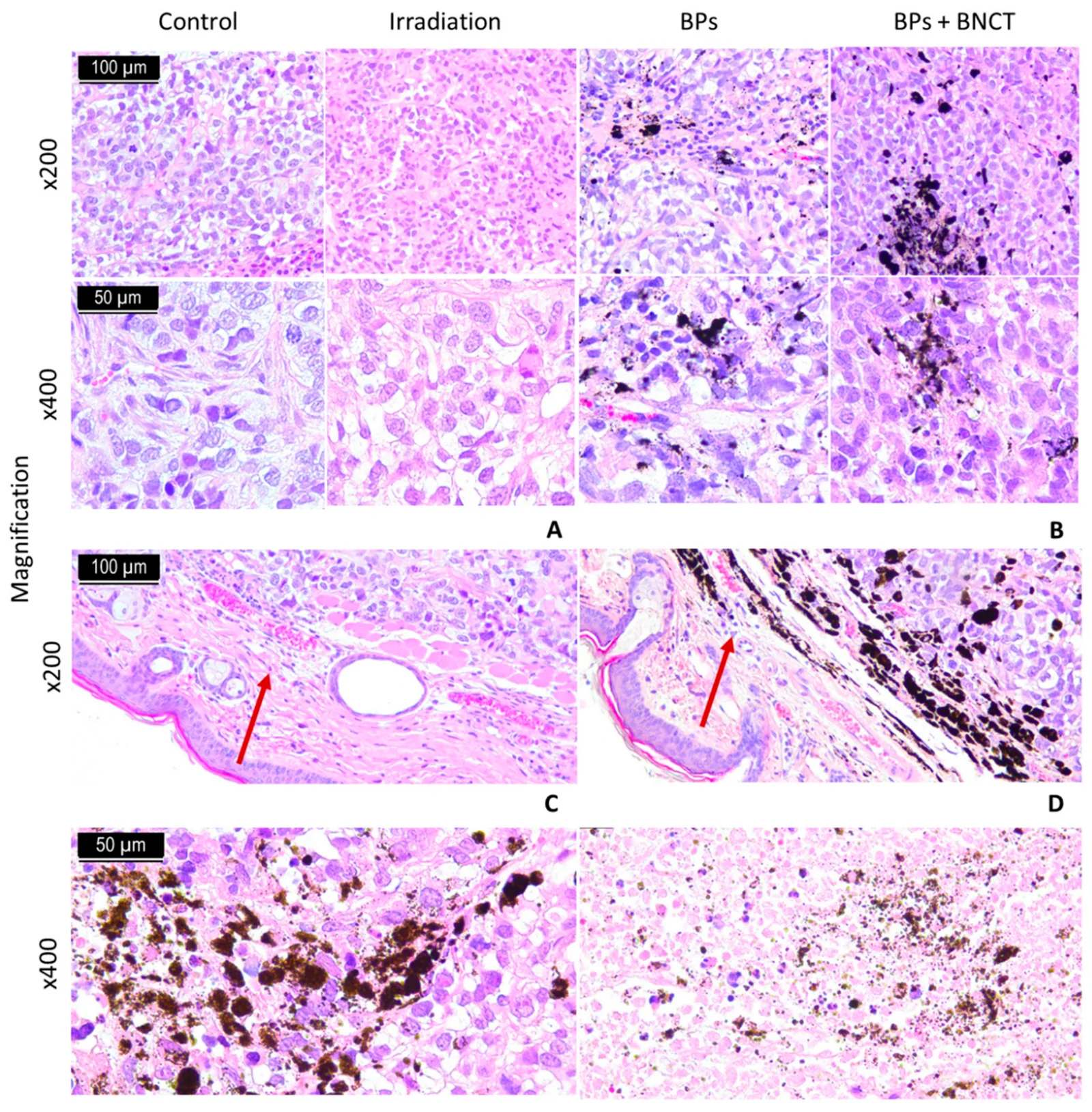
Microstructure of the BT-474 xenograft in female SCID mice after injection of BPs. Magnification: ×200 and ×400. (**A**,**B**) Border between the subcutaneous fat tissue (indicated by arrows) and tumor cells: (**A**) control, (**B**) after treatment only by BPs. Distribution of nanoparticles in tumor tissue: (**C**) proliferating cells, (**D**) necrotic areas.

Histological examination of the lungs, spleen, heart, and brain of female mice removed from the experiment between weeks 7 and 10 did not reveal any metastases, nanoparticles or other abnormalities (Figure 9). In contrast, the examination of BT-474 xenografts in female mice after the injection of the nanoparticles showed (Figure 10) that the BPs partially accumulated in the subcutaneous fat adjacent to the xenograft (Figure 10B), but particularly large amounts were found inside the xenograft, where the BPs formed large aggregates. These aggregates were observed in areas with actively proliferating tumor cells (Figure 10C), whereas in regions of coagulation necrosis, the aggregates of BPs were small and fragmented (Figure 10D).

## 4. Discussion

Successful BNCT applications have been reported for different types of tumors [27], including glioma [10], colon carcinoma [19], hepatocellular carcinoma [28], and breast carcinoma [29]. However, most successful BNCT results have been obtained using BPA and BSH as boron delivery agents, both of which have several disadvantages. Numerous attempts to create alternative boron delivery agents that meet the requirements of BNCT have not been successful due to the complexity of synthesis, insufficient tumor selectivity, or toxicity [14,30]. Moreover, despite active research into new boron delivery approaches for BNCT, including the use of nanoparticles, high BNCT efficacy has not yet been achieved [31].

One promising approach involves the use of functionalized boron carbide nanoparticles, which accumulate in high concentrations in tumor tissue [32]. Another example is the use of functionalized polymer particles loaded with boron-containing agents [33]. However, a limitation of this approach is the lack of convincing evidence of its in vivo efficacy. The use of elemental boron nanoparticles with targeted delivery to tumor cells represents a promising direction in BNCT. These agents offer several advantages. On the one hand, they have a high density of boron atoms (~10^2^–10^5^ atoms per particle), maximum potential for active delivery due to the functionalization of their surface with targeting molecules and relatively low production cost. The results of in vitro BNCT studies in the presence of the tested nanoparticles for human breast adenocarcinoma BT-474 showed high efficacy. MTT-test and clonogenic assay data convincingly demonstrated that BNCT with BPs at a dosage of 40 μg/mL equally effectively slowed down cell growth and inhibited their colony-forming activity (Figure 5 and Table 1). For comparison, according to previous studies, incubation of T98G human glioma cells with the same concentration of boron nanoparticles resulted in a clonogenic activity decrease by only 15.5% after BNCT [34].

Studies on SCID mice with BT-474 human breast tumor xenografts largely confirmed the findings from the in vitro experiments. It was found that BNCT with BPs inhibited the growth of xenografts (Table 2), but only during the first few weeks after irradiation. Xenograft growth in animals after BNCT was significantly slowed down compared to the control animals starting from day 17, and compared to the irradiated animals starting from day 39 after irradiation (Figure 7). The inhibition of xenograft growth in animals after BNCT relative to the control animals was observed as early as day 7 (Table 2) and reached of 78% by the end of the experiment. In contrast, in the mice exposed to radiation alone the TGI did not exceed 49%, respectively, throughout the experiment. The results are also supported by the animal survival rates shown in the Kaplan–Meier curves (Figure 8). For the animals from BNCT with BPs group, 100% survival was noted on day 70, when the experiment was stopped.

The reduction in xenograft size in animals exposed to neutrons without boron preparations was likely due to the presence of fast neutron and gamma radiation components in the beam, which had a detrimental effect on the cells. However, it has been shown that the epithermal neutron flux obtained from an accelerator-based neutron source at therapeutic doses is relatively safe for normal tissues and does not affect the viability of laboratory animals, nor does it cause any significant damage to vital organs or skin [35].

The histological studies of xenografts from different groups did not reveal any significant differences in the tumor structure. In the groups with the introduction of nanoparticles, their successful accumulation in xenografts was detected (Figure 10). At the same time, it is known that the success of BNCT is determined by the ability of boron to penetrate cells [14]. Also, despite some loss of injected particles in the subcutaneous layer, most of them were delivered to deeper layers of the tumor tissue (Figure 10A,B). The studied nanoparticles did not accumulate in normal tissues for prolonged periods and did not cause any structural damage (Figure 9).

## 5. Conclusions

The results demonstrated the high efficacy of nanoparticles obtained by laser fragmentation of boron micropowder with functionalization by affibody ZHER2:342 targeted the HER2 for of BNCT in the both in vitro experiments with HER2 positive BT-474 cells and in vivo studied of SCID mice with xenografts of human breast adenocarcinoma BT-474.

## Figures and Tables

**Table 1:** Results of the clonogenic test for BT-474 human breast tumor cells after the addition of the studied BPs and following BNCT.

Group	Control	Irradiation	BPs	BPs + BNCT
M ± SE	57.0 ± 3.8	54.3 ± 3.0	35.3 ± 2.0	0
Proportion, %	100	95	62	0
The view of the cells in the well	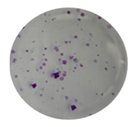	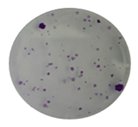	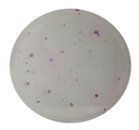	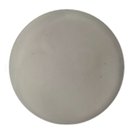

**Table 2:** Weekly TGI values in female SCID mice with xenografts of BT-474 human breast tumor cells after BNCT with intratumoral administration of BPs.

Group/TGI, %	1 Week	2 Week	3 Week	4 Week	5 Week	6 Week	7 Week	8 Week
Irradiation	25	42	34	44	49	35	30	42
BPs	22	6	–15	–5	1	–31	–24	
BPs + BNCT	27	49	56	65	72	75	72	78

## Data Availability

The original contributions presented in this study are included in the article. Further inquiries can be directed to the corresponding author.
